# Supplementary material for: A machine learning assistant for detecting fraudulent activities in synchronous online programming exams
Source: PeerJ Comput Sci. 2025 Sep 9;11:e3159. doi: 10.7717/peerj-cs.3159 (PMC12453727; doi:10.7717/peerj-cs.3159)
Supplement: Supplemental Information 1 [file peerj-cs-11-3159-s001.zip › assistant/src/main/resources/VeyonHD.pdf]

Veyon Master

Monitoring | Fullscreen demo | Window demo | Lock | Remote view | Remote control | Power on | Reboot | Power down | Log in | Log off | Text message | Run program | Open website | File transfer | Screenshot | Record

Archivo Editar Ver Git Proyecto Compilar Depurar Prueba Analizar Herramientas Extensiones Ventana Ayuda Buscar codeanalysis-web

Debug | Any CPU | http | GitHub Copilot

AnalysesController.cs | SignUpResult.cshtml | SignUp.cshtml | Login.cshtml | SignUpModel.cs | LoginModel.cs | UsersService.cs | Program.cs | ProgramsController.cs | AuthController.cs | site.css | appsettings.json | ResultssService.cs

codeanalysis-web | codeanalysis_web.Controllers.AnalysesController | _analysesService

```
        }

        return RedirectToAction("Error", "Home", new { statusCode = (int)result.StatusCode, message = result.Content });
    }

    [Authorize]
    [HttpGet]
    0 referencias | 0 cambios | 0 autores, 0 cambios
    public async Task<IActionResult> Create()
    {
        var result = await _categoriesService.GetCategories();
        if (result.IsSuccessful)
        {
            string[] categories = JsonConvert.DeserializeObject<string[]>(result.Content);
            ViewBag.Categories = new SelectList(categories.OrderBy(x => x));
        }
        else
        {
            return RedirectToAction("Error", "Home", new { statusCode = (int)result.StatusCode, message = result.Content });
        }

        result = await _syntacticConstructsService.GetSyntacticConstructs();
        if (result.IsSuccessful)
        {
            string[] syntacticConstructs = JsonConvert.DeserializeObject<string[]>(result.Content);
            ViewBag.SyntacticConstructs = new SelectList(syntacticConstructs.OrderBy(x => x));
        }
        else
        {
            return RedirectToAction("Error", "Home", new { statusCode = (int)result.StatusCode, message = result.Content });
        }
        return View(new CreateAnalysisModel() { RDN = ReverseMail(User.FindFirstValue(ClaimTypes.Email)).ToLower() + ".analyses." });
    }

    [Authorize]
    [HttpPost]
    0 referencias | uo296434, Hace 225 días | 1 autor, 1 cambio
    public async Task<IActionResult> Create(CreateAnalysisModel model)
    {
        if (ModelState.IsValid && model.IsValid())
        {
```

121 % | 0 | 33 | Línea: 12 | Carácter: 19 | SPC | CRLF

Explorador de pruebas | Lista de errores | Salida | Consola del Administrador de paquetes | PowerShell para desarrolladores

Listo | 0 / 0 | 6 | ines | codeanalysis

ESP 20:13 31/01/2025

**TOM SMITH - UO157632**

Locations & computers | Screenshots | Search users and computers

15:38
