# Supplementary figures and images for: A machine learning assistant for detecting fraudulent activities in synchronous online programming exams
Source: PeerJ Comput Sci. 2025 Sep 9;11:e3159. doi: 10.7717/peerj-cs.3159 (PMC12453727; doi:10.7717/peerj-cs.3159)

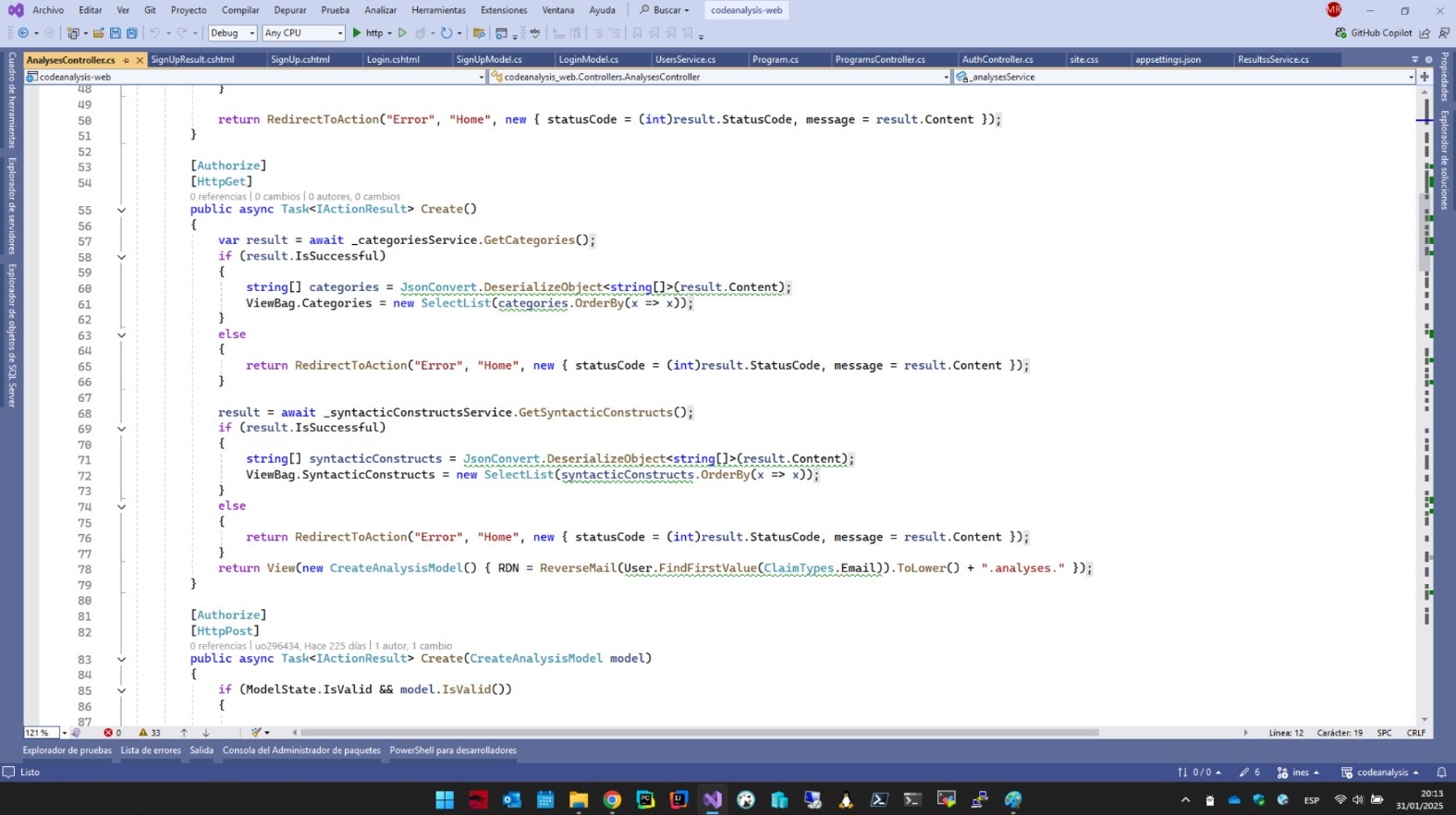

Supplement: Supplemental Information 1 [file peerj-cs-11-3159-s001.zip › assistant/src/main/resources/imagen-original.jpeg]

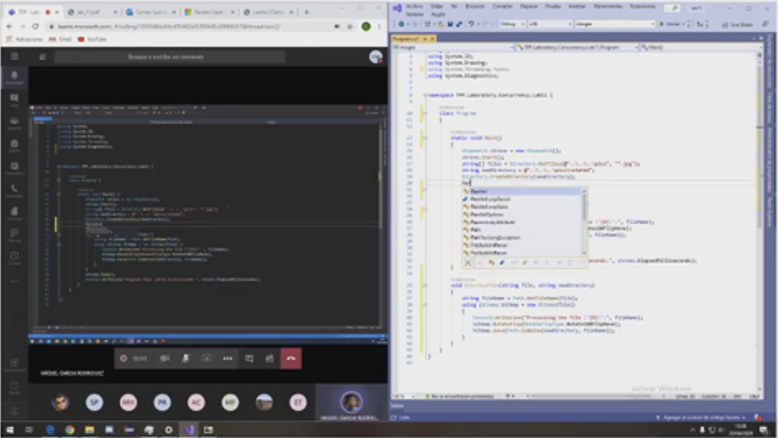

Supplement: Supplemental Information 1 [file peerj-cs-11-3159-s001.zip › assistant/src/main/resources/img1-old.png]

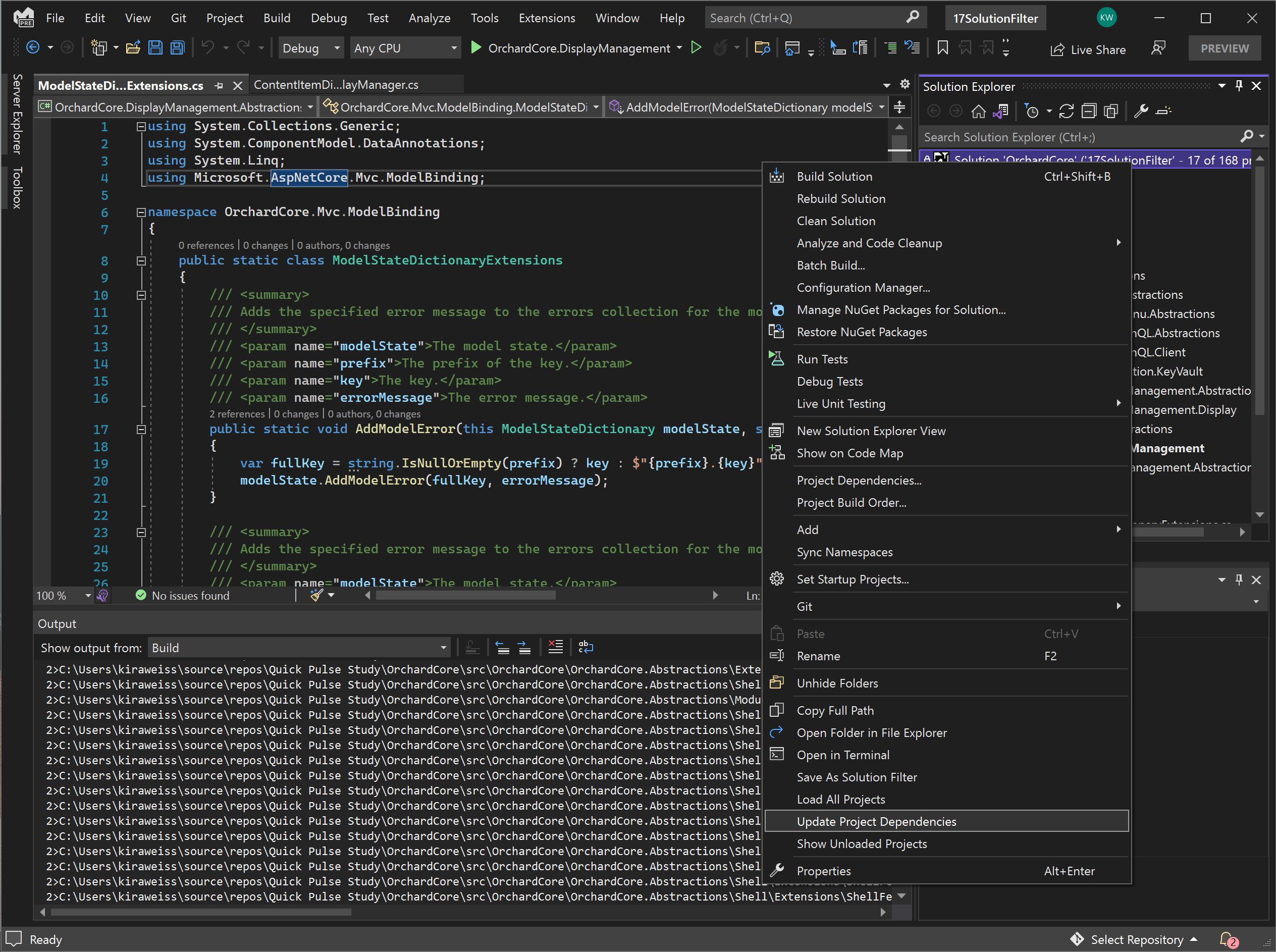

Supplement: Supplemental Information 1 [file peerj-cs-11-3159-s001.zip › assistant/src/main/resources/img1-big.png]

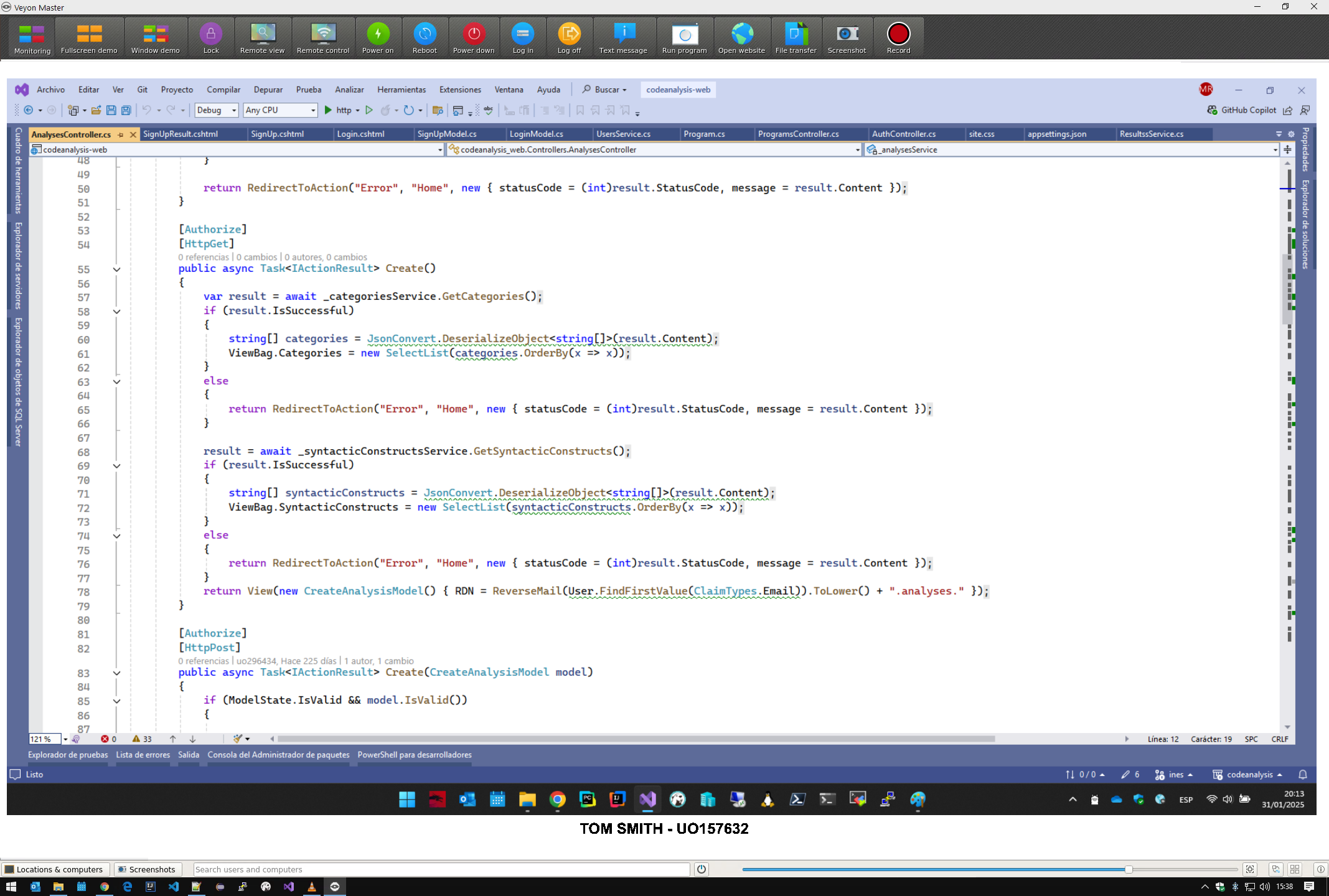

Supplement: Supplemental Information 1 [file peerj-cs-11-3159-s001.zip › assistant/src/main/resources/VeyonHD.png]

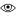

Supplement: Supplemental Information 1 [file peerj-cs-11-3159-s001.zip › assistant/src/main/resources/eye.png]

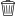

Supplement: Supplemental Information 1 [file peerj-cs-11-3159-s001.zip › assistant/src/main/resources/bin.png]

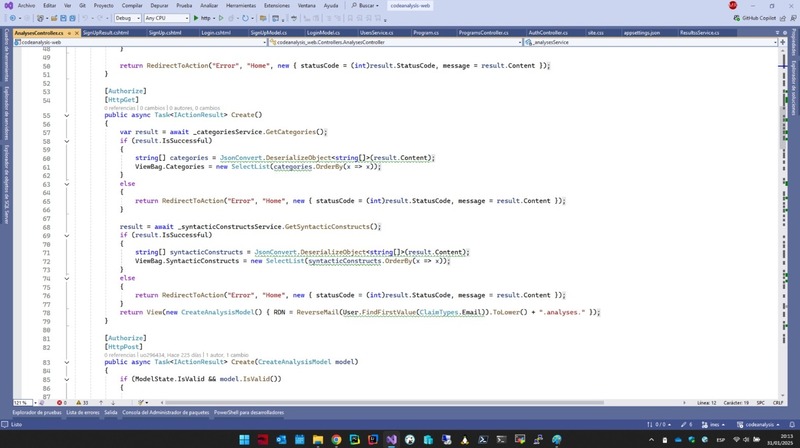

Supplement: Supplemental Information 1 [file peerj-cs-11-3159-s001.zip › assistant/src/main/resources/img1.jpg]

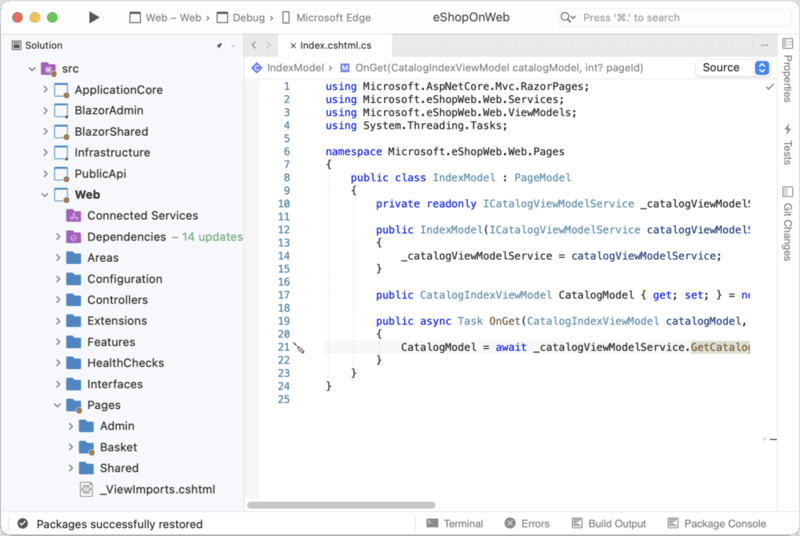

Supplement: Supplemental Information 1 [file peerj-cs-11-3159-s001.zip › assistant/src/main/resources/img1.png]

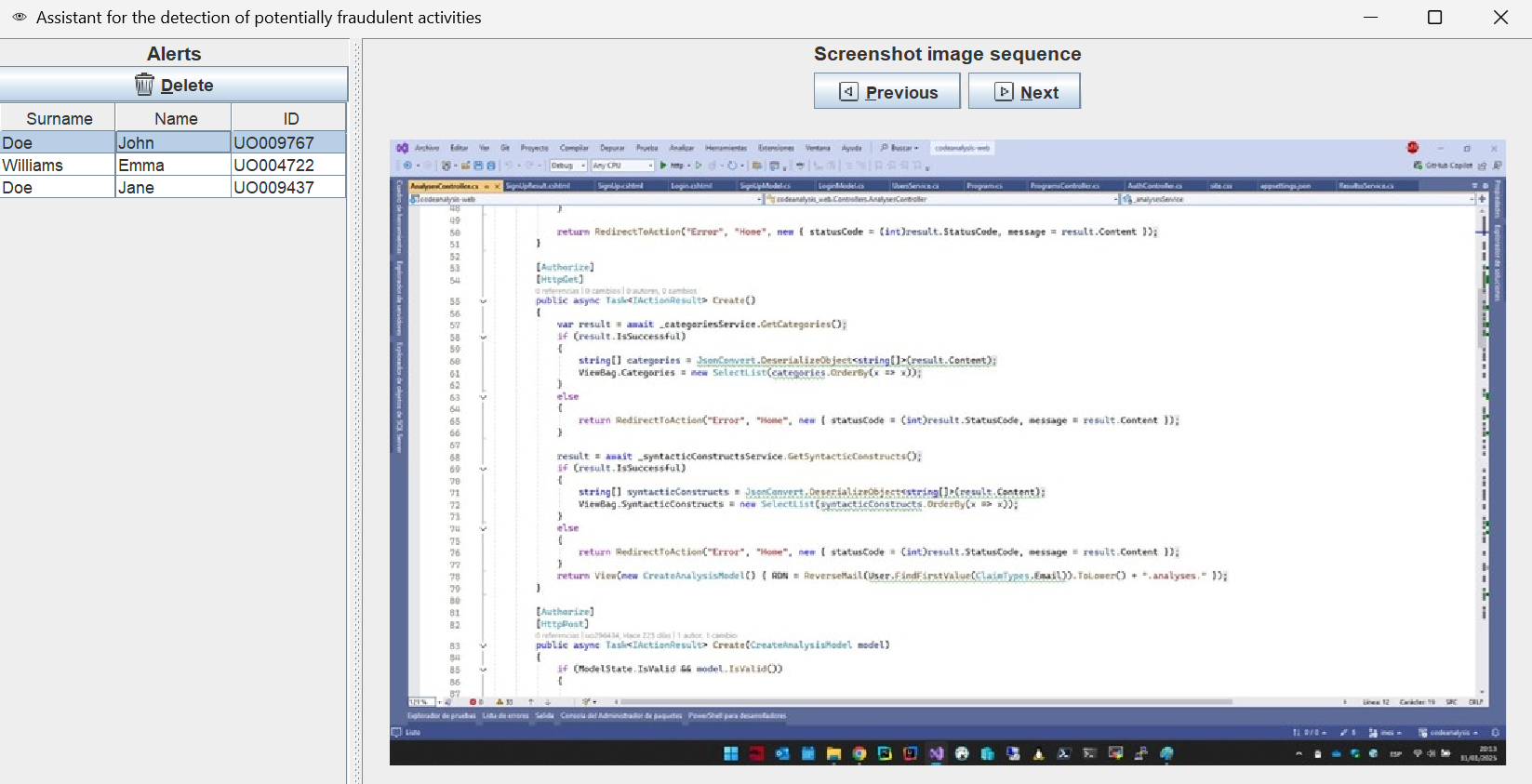

Supplement: Supplemental Information 1 [file peerj-cs-11-3159-s001.zip › assistant/src/main/resources/captura-assistant-2.png]

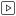

Supplement: Supplemental Information 1 [file peerj-cs-11-3159-s001.zip › assistant/src/main/resources/next.png]

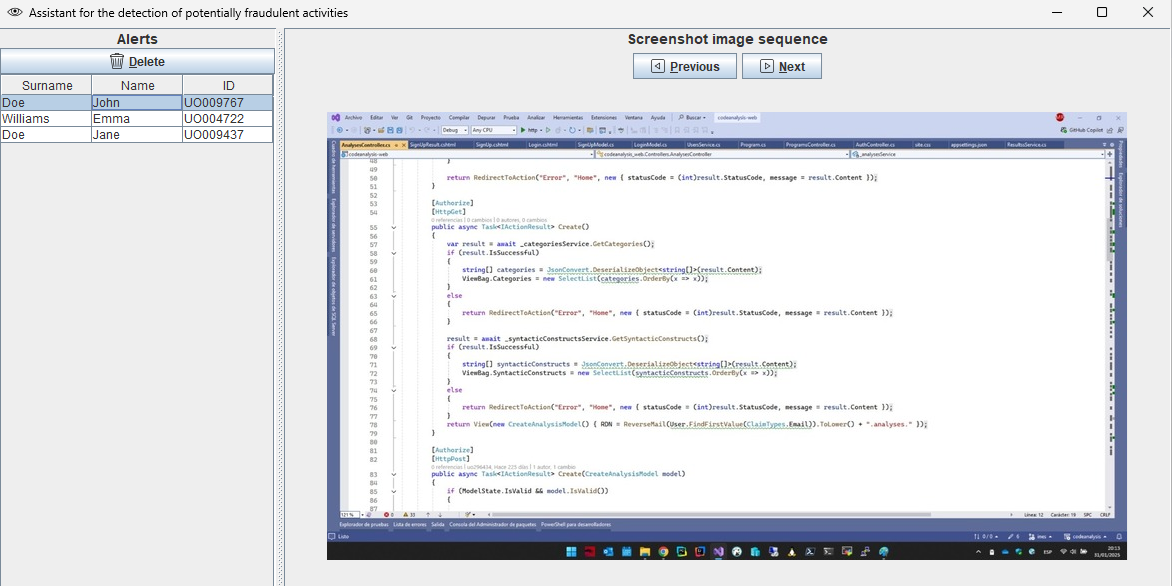

Supplement: Supplemental Information 1 [file peerj-cs-11-3159-s001.zip › assistant/src/main/resources/captura-assistant-1.png]

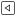

Supplement: Supplemental Information 1 [file peerj-cs-11-3159-s001.zip › assistant/src/main/resources/previous.png]
